# Dose optimisation for intraoperative cone-beam flat-detector CT in paediatric spinal surgery

**DOI:** 10.1007/s00247-012-2396-0

**Published:** 2012-06-06

**Authors:** Asger Greval Petersen, Søren Eiskjær, Jon Kaspersen

**Affiliations:** 1 Department of X-ray Physics, Region of Northern Jutland, Broenderslev, Denmark; 2 The Spinal Unit, Department of Orthopaedic Surgery, Aalborg University Hospital, Aalborg, Denmark; 3 Merkurvej 13, Postboks 183, 9700 Brønderslev, Denmark

**Keywords:** O-arm, Paediatric spine, Preoperative CT scan, Radiation dose

## Abstract

**Background:**

During surgery for spinal deformities, accurate placement of pedicle screws may be guided by intraoperative cone-beam flat-detector CT.

**Objective:**

The purpose of this study was to identify appropriate paediatric imaging protocols aiming to reduce the radiation dose in line with the ALARA principle.

**Materials and methods:**

Using O-arm® (Medtronic, Inc.), three paediatric phantoms were employed to measure CTDI_w_ doses with default and lowered exposure settings. Images from 126 scans were evaluated by two spinal surgeons and scores were compared (Kappa statistics). Effective doses were calculated. The recommended new low-dose 3-D spine protocols were then used in 15 children.

**Results:**

The lowest acceptable exposure as judged by image quality for intraoperative use was 70 kVp/40 mAs, 70 kVp/80 mAs and 80 kVp/40 mAs for the 1-, 5- and 12-year-old-equivalent phantoms respectively (kappa = 0,70). Optimised dose settings reduced CTDI_w_ doses 89–93%. The effective dose was 0.5 mSv (91–94,5% reduction). The optimised protocols were used clinically without problems.

**Conclusions:**

Radiation doses for intraoperative 3-D CT using a cone-beam flat-detector scanner could be reduced at least 89% compared to manufacturer settings and still be used to safely navigate pedicle screws.

## Introduction

It is well-documented that radiographic examinations for spinal deformity during childhood increase the lifetime risk of cancer, particularly breast cancer [1, 2]. It follows that precautions need to be taken to reduce radiation burden.

New technologies have advanced surgery for spinal deformities. The use of pedicle screws requires accurate placement to avoid damage to the spinal cord and the large vessels in front of the spine. To secure the correct placement of pedicle screws, intraoperative imaging is imperative. For many years, fluoroscopy was the only available intraoperative imaging modality. However, in the last two decades, navigation based on preoperative CT has been developed. However, it is not widely used, possibly due to the time-consuming registration process (coupling of the preoperative CT with patient anatomy) of up to 15–20 min per vertebra making it almost impossible to use in spine deformity surgery with instrumentation of 10–15 vertebrae in a single operation. On the other hand, the need for navigation is most pressing in young patients who have both deformed and small pedicles.

Intraoperative cone-beam flat-detector X-ray systems have changed spinal surgery and are rapidly being implemented worldwide. These provide both 2-D fluoroscopic and 3-D images, which when coupled to a navigation system add significant value to surgical outcomes [3–6]. With such a system, the registration process is fast and navigation can be performed in four to six vertebrae before a new 3-D scan is needed. The drawbacks are the price (scanner and essential navigation system, approximately €700,000 in 2012) and the increased radiation burden.

According to Zhang et al. [7], the patient dose delivered by the scanner in default mode is equivalent to 0.5–0.6 times the dose delivered by a conventional 64-slice CT-scanner. Usually at least two to three scans are required during surgery for a spinal deformity. Other authors [8, 9] have said the resultant dose is associated with increased cancer risk. On the basis of estimates from the United States [8], it is thought that in children younger than 15 years approximately 80–90 per 100,000 CT examinations attribute to cancer mortality.

Our aim was to identify appropriate intraoperative exposure settings for children for a cone-beam flat-detector system, aiming to reduce the radiation dose.

## Materials and methods

### Scanner

The O-arm® (Medtronic, Inc.) cone-beam flat-detector system was used. The system has an O-ring type gantry and an X-ray tube equivalent to 32 kW, and a X-ray filter of 4 mm Al. The flat-panel has an amorphous silicon-based detector of 30 cm × 40 cm with a 0.194-mm pixel pitch. The system can be configured in 2-D fluoroscopic mode or 3-D mode. In this study, only 3-D mode was used and only the low-definition mode. In the low-definition mode, 192 single images (compared to 392 images in high-definition mode) of slice thickness 0.833 mm were recorded in a 360-degree rotation of the detector and the radiation source with an image matrix of 512 × 512. The time for image acquisition was 13 s with a beam on-time of 3.91 s. The source-to-isocentre distance of the O-arm was 64.7 cm and the source-to-detector distance 116.8 cm. The collimated X-ray beam was 22.18 × 16.62 cm and the 3-D reconstructed volume was 20 cm × 15 cm.

Besides the 16 default imaging protocols (head, chest, abdomen and extremity applied to small, medium, large and extra large patients, respectively), the O-Arm allows manual adjustment of kVp and mA. With the manual setting, the kVp can be varied between 50 and 120 with 1-kVp intervals, and the mA can be varied between 10 and 120 in predefined steps.

The manufacturer’s advice (personal communication) for abdominal imaging is to use the default values for small (waist circumference 12–26 cm) or the default values for medium (waist circumference 20–34 cm).

### Phantoms

**Fig. 1:**
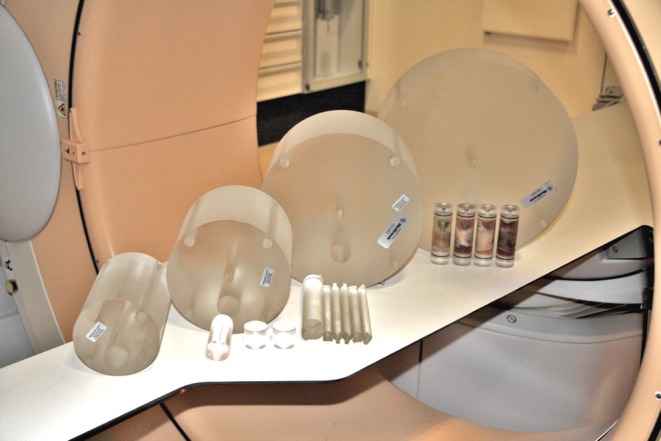
Polymethyl-methacrylate (PMMA) phantoms with diameters 10–32 cm that were scanned together with inserts of bone specimens, solid PMMA rods and a tungsten wire

**Fig. 2:**
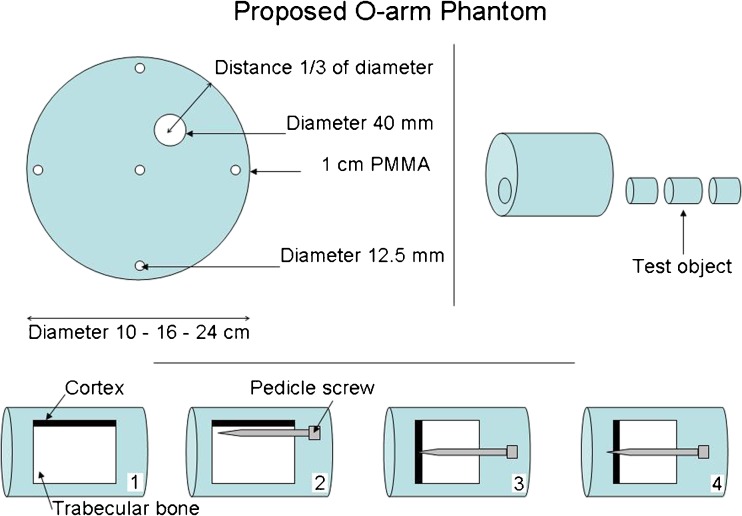
Design of both polymethyl-methacrylate (PMMA) cylinders and the four inserts of bone bank bones from patients having undergone hip arthroplasty and pedicle screws: (*lower inserts from left to right*) pure bone, screw parallel to cortex, screw just reaching the cortex, screw just penetrating cortex

The studies were conducted using polymethyl-methacrylate (PMMA) phantoms to estimate patient dose equivalence. Four cylindrical PMMA phantoms were made with diameters of 10 cm, 16 cm, 24 cm and 32 cm (Fig. 1). The 10-cm PMMA would be equivalent to the body of a child <1 year old, and the phantoms of 16 cm, 24 cm and 32 cm would be equivalent to the body of a 5-year old, 12-year-old and an adult, respectively [3, 10]. All phantoms were 15 cm long and had standard holes for CTDI dosimetry at the centre and at the perimeter but were also made with a 3-cm cylindrical hole through the phantom in a radial distance from the perimeter corresponding with the position of the spine in a patient of the same size. In this hole, four samples made from human femoral neck (Fig. 2), three of which also contained pedicle screws, were placed and scanned one at a time in the centre of the phantom with the remaining holes filled with solid PMMA rods. The locations of the screws with respect to the cortical edge of the bone were chosen to match clinically realistic scenarios.

### Dose measurements

The dose to the patient was expressed as CTDI_w_ and calculated from the dose measured in the PMMA cylinders in the periphery and centre with a 100-mm pencil ion chamber with all other holes filled with solid PMMA rods. The CTDI_w_ was measured for each phantom size using both the default and the optimised values for the setting of kVp and mA. The absorbed doses of radiosensitive organs and total effective doses were calculated for each phantom size, using both the default and the optimised values for kVp and mAs. The calculations were performed with the PCXMC Monte Carlo dose simulation program version 2.0 (STUK - Radiation and Nuclear Safety Authority, Helsinki, Finland) using the ICRP 103 tissue weighting-factors [11]. To perform this calculation, the PCXMC program requires measurements of the incident air kerma at the surface of each phantom without backscatter factor. This was measured at the isocentre with the 100-mm pencil ion chamber and calculated for the surface of each phantom using the inverse square law. To reduce the computation time with PCXMC, the 360-degree arc was divided into 12 parts of 30 degrees. The absorbed doses to the radiosensitive organs and total effective doses were calculated by summing the contributions of each of the 12 dose calculations.

### Image quality assessment

Signal-to-noise ratio (SNR) and contrast-to-noise ratio (CNR) were measured. SNR was calculated as S1/σ1, where S1 represents the mean pixel value within a region of interest (ROI) in the PMMA phantom with all PMMA rods inserted at the position of the bone samples and σ1 represents the standard deviation of pixel values within the same ROI. CNR was estimated as |S2−S1|/σ1, where S2 is the mean pixel value in the pure bone sample. For this purpose, ImageJ version 1.32 (http://rsb.info.nih.gov/ij/) was used.

The subjective image quality of all phantom images with pedicle screws (126 scans) was evaluated independently by two spinal surgeons, both with more than 10 years of experience in spinal surgery and intraoperative imaging. The image quality was deemed adequate if the outlines of the bones were visible and if it was possible to discern whether the screw penetrated the bone. If the outlines of the bone sample and the screw position could be visualised with certainty, the image quality was deemed as adequate. In all other cases, the image quality was deemed inadequate. Interobserver agreement was measured with Kappa statistic [12, 13].

### Dose optimisation

The scans started with the factory default settings, followed by a series of scans with decreasing mA until the lowest possible tube current of 10 mA was reached. If image quality was still acceptable, additional scans were acquired at 10 mA while reducing kV until an unacceptable image quality was achieved. The scan parameters with the lowest dose to the phantom where the image quality in all four bone samples were accepted were recorded as the suggested optimum low-dose settings for that specific phantom size. Scans were then acquired with all holes filled with solid PMMA rods to measure image noise and dose.

After institutional review board approval, the recommended new low-dose 3-D spine protocol was tested in clinical practice in 15 children (10 females) with severe deformities in whom it would not have been possible to place pedicle screws without navigation. This would otherwise require conventional preoperative CT with a higher dose. Only the 16-cm and 24-cm protocols were used. The average age was 11.5 years (range, 2–17 years). An average of 9.7 vertebrae were scanned (range, 3–16) using an average of 2.5 acquisitions (range, 1–4), as shown in Table 1.

**Table 1:** Patient characteristics, number of scans, protocols and radiation doses for 15 children undergoing corrective spinal surgery using a cone-beam scanner with radiation-dose optimised exposure for guiding pedicle screw placement

Patient	Sex; age at surgery (years)	Diagnosis	Number of levels	Patient height (cm)/weight (kg)	Number of intraoperative scans	DLP mGy·cm^a^	Scan protocol exposure settings kVp/mA	Instrumentation
1	M, 17	Idiopathic scoliosis	12	185/76	3	94	80/20	Conventional^b^
2	F, 2	Hemivertebra	3	82/11	1	17	80/10	Conventional^b^
3	M, 13	Neuromuscular scoliosis	16	159/31	4	80	70/20	Conventional^b^
4	F, 15	Idiopathic scoliosis	7	158/45	2	65	80/20	Conventional^b^
5	F, 15	Idiopathic scoliosis	9	162/52	2	40	70/20	Conventional^b^
6	M, 12	Neuromuscular scoliosis	15	152/32	3	60	70/20	Conventional^b^
7	M, 6	Infantile scoliosis	4	99/13	2	65	80/20	Growth rod
8	F, 5	Infantile scoliosis	8	109/16	3	86	80/10	Conventional^b^
9	F, 14	Idiopathic scoliosis	6	161/48	2	40	70/20	Conventional^b^
10	F, 12	Neuromuscular scoliosis	13	121/28	4	80	70/20	Conventional^b^
11	F, 11	Neuromuscular scoliosis	11	150/43	2	60	80/10,70/20	Conventional^b^
12	M, 14	Neuromuscular scoliosis	16	165/40	4	80	70/20	Conventional^b^
13	F, 14	Idiopathic scoliosis	10	175/75	2	40	70/20	Conventional^b^
14	F, 8	Spondylolisthesis gr. 4	4	134/28	1	16	80/10	Conventional^b^
15	F, 14	Idiopathic scoliosis	11	171/60	3	97	80/10	Conventional^b^

^a^ According to the manufacturer

^b^ Conventional segmental instrumentation with pedicle screws and rods

## Results

**Fig. 3:**
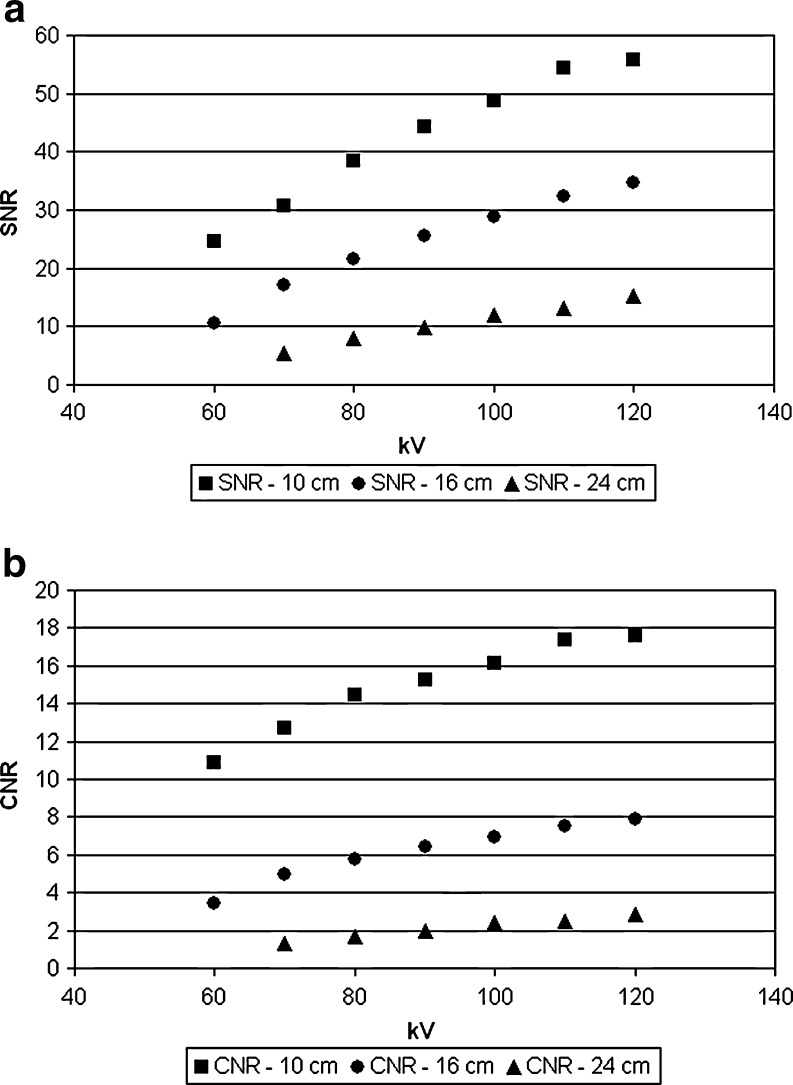
Objective image quality parameters for different phantom diameters (10 cm, 16 cm and 24 cm) at constant tube current. **a** Signal-to-noise ratio (SNR) against kV. **b** Contrast-to-noise ratio (CNR) against kV

As expected, SNR and CNR decreased nonlinearly with decreasing radiation dose (Fig. 3). SNR and CNR at lowest acceptable dose decrease with increasing phantom size. Therefore, patient size is a crucial factor when choosing the values of SNR and CNR for lowest acceptable image quality.

Table 2 compares the preset and optimised protocols and dose values. For both the 10-cm and the 16-cm phantoms, the optimised dose was compared with the dose of the standard small chest protocol. For the 24-cm phantom, the optimised dose was compared with dose of the standard medium chest protocol. All optimised settings yielded at least a 89% reduction in dose, with the 10-cm protocol yielding a 93% dose reduction as shown in Tables 2 and 3. This was corroborated when the effective dose was calculated with effective dose values approximately 0.5 mSv for the optimised protocols and effective dose values 12–19 times higher for the default protocols as shown in Table 4.

**Table 2:** Radiation doses at default exposure settings for different sizes of body/anatomy (*L* large, *M* medium, *S* small) and at exposures optimised for low-dose 3-D paediatric spine imaging with dose reduction factors for all phantom sizes

Phantom diameter (cm)	Protocol	kV	mA	mAs	CTDI_w_ (mGy)	Dose reduction, %
32	L	120	50	200	11.3	
24	M	120	40	160	14.5	
24	Low dose	70	20	80	1.6	89.2
16	S	120	32	128	14.4	
16	Low dose	80	10	40	1.5	89.4
10	S	120	32	128	16.6	
10	Low dose	70	10	40	1.2	93.0

**Table 3:** Scan protocols for a 1-year-old-equivalent phantom with dose reduction achieved and scoring of image quality

Phantom diameter (cm)	Scan protocol	kV	mA	mAs	CTDI_w_ (mGy)	Dose reduction, %	Image quality scoring
10	1	120	32	128	16.59	0	OK
10	2	120	20	80	10.41	37.3	OK
10	3	120	10	40	5.47	67.0	OK
10	4	100	10	40	3.39	79.6	OK
10	5	80	10	40	1.81	89.1	OK
10	6	70	10	40	1.15	93.1	OK
10	7	60	10	40	0.69	95.8	Not OK

**Table 4:** Estimated effective doses at default exposure settings for different sizes of body/anatomy (*L* large, *M* medium, *S* small) and at exposures optimised for 3-D paediatric spine imaging with dose reduction factors for all phantom sizes

Phantom diameter (cm)	Protocol	kV	mA	mAs	Effective dose (mSv)	Dose reduction, %
24	M	120	40	160	8.3	
24	Low dose	70	20	80	0.4	94.7
16	S	120	32	128	6.3	
16	Low dose	80	10	40	0.5	91.7
10	S	120	32	128	5.8	
10	Low dose	70	10	40	0.5	92.2

**Fig. 4:**
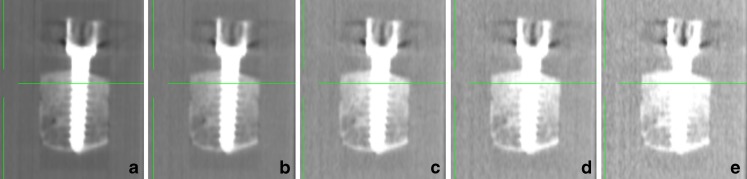
Scan of a 10-cm polymethyl-methacrylate phantom with bone insert at default exposure settings 120 kVp/128 mAs (**a**), and at 120kVp/40mAs (**b**), 80kVp/40mAs (**c**), at the lowest exposure for acceptable image quality, 70 kVp/40 mAs (**d**) and below acceptable level at 60 kVp/40 mAs (**e**)

The observers agreed that the lowest acceptable dose for intraoperative imaging was 70 kVp/40 mAs for the 1-year-old-equivalent phantom (10 cm), 70 kVp/80 mAs for the 5-year-old-equivalent phantom (16 cm) and 80 kVp/40 mAs for the 12-year-old-equivalent phantom (24 cm). Figure 4 shows examples of images of pedicle bone implants with minimal penetration at different dose settings.

The interobserver agreement for all scans had a kappa = 0,70 (substantial agreement [14]). For the 10-cm, 16-cm and 24-cm phantoms, kappa was 0.64, 0.72 and 0.70, respectively, also indicating substantial agreement.

**Fig. 5:**
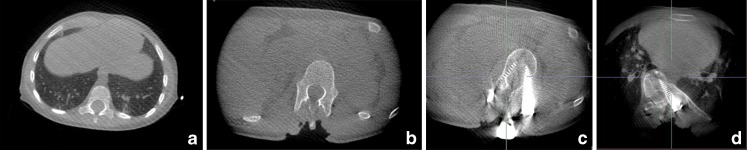
Examples of intraoperative images with different dose settings with and without pedicle screws. **a** In a 2-year-old boy using 80 kVp/40 mAs (16-cm protocol). **b** In a 14-year-old girl using 70 kVp/40 mAs (24-cm protocol). **c** In a 14-year-old girl using 70 kVp/40 mAs (24-cm protocol), there are moderate to severe streak artifacts. **d** In a 17-year-old boy at 80 kVp/80 mAs, there is decreased metal artifacts compared to (**c**) at approximately 30% higher dose

In all 15 operations using the optimised setting, the spinal surgeon achieved adequate intraoperative imaging with the cone-beam flat-detector scanner followed by navigation using the Stealth system (Stealth Station®, Medtronic, Minneapolis, USA). Figure 5 shows examples of intraoperative images with different dose settings with and without pedicle screws. The same protocol was used both for the thoracic and abdominal regions. In none of the cases was control CT done postoperatively because of suspected misplacement of screws or neurological deterioration. Conventional radiographs showed satisfactory placement of all screws and the neurological status was unchanged for all children. Spinal cord monitoring (sensory evoked potentials (SEP) and motor evoked potentials (MEP)) did not indicate damage to the spinal cord or the spinal nerves in any of the children.

## Discussion

The phantom model allowed a systematic evaluation of different dose settings from the highest to the lowest possible. It also allowed different bone inserts containing pedicle screws to be evaluated with regard to image quality. This is especially important as the cornerstone of image guidance during pedicle screw insertion is the ability to clearly identify the cortex relative to the pedicle screw. Streak artefacts from metallic implants are a major concern, and any realistic model needs to incorporate this. Our phantom model could only be used to evaluate 90-degree perforations of the cortex due to the small size of the bone samples. However, to compensate for this limitation, for the sample with the perforating screw, the perforation was made as small as possible.

The CTDI_100_ does not measure the actual absorbed dose by the individual patient but should be considered an index for comparisons. The homogeneous PMMA does not simulate the different tissue types and heterogeneities in vivo. The CTDI_100_ will underestimate the radiation dose because the 100-mm pencil ion chamber only partly covers the collimated beam width. For X-ray beams wider than 40 mm, a pencil chamber longer than 100 mm is required. The CTDI_w_ will underestimate the ideal CTDI_w_ with approximately 20% at a collimated beam width of 20 mm [15]. Dixon [16] has described an alternative method to directly measure the dose at the central scan plane using a small ion chamber.

The patient dose of the cone-beam system relative to CT has been described by Zhang et al. [7], who showed that with identical techniques (kVp, mAs, etc.) and with the same scan length, the cone-beam system in 3-D mode delivered approximately half the radiation dose of a 64-slice CT scanner. Their use of a Farmer ion chamber gave a more correct estimation of patient dose compared to the 100-mm ion chamber used in our study for dose comparison as it could be used to directly measure the point dose at the centre of the scan length. Differences between cone-beam systems and CT are to be expected due to differences in source-to-isocentre distance (SID). The SID of the cone-beam system was 64.7 cm while the SID of the 64-slice CT scanner in question was 57.1 cm. This accounts for approximately 29% of the dose difference [7]. Second, there is a difference in fan beam angle. The cone-beam system has an angle of approximately 20 degrees, and the CT scanner approximately 45 degrees. This leads to less peripheral radiation dose measurements for the cone-beam system because the ion chamber is outside the beam during parts of the scan [7]. The differences in over beaming between the cone-beam system and the CT scanner will account for a slight dose reduction in favour of the cone-beam system [7].

Would a vertebra have been a better bone sample for the phantom part of the study? This would without doubt have better represented the normal anatomy. The cortical thickness of the femoral head is greater than the cortical thickness of the vertebra, and this would have influenced the accuracy of the imaging representation [17]. A more accurate model would probably improve the surgeons’ ability to discern whether a screw was placed correctly. The bone mineral density of children and adolescents changes significantly with age [18, 19]. The bone samples used here were acquired from the local bone bank. The femoral necks used in this study were obtained solely from patients undergoing hip arthroplasty, and these patients are in general not osteoporotic [18, 21]. Mean BMD for a hip arthroplasty patient is approximately 1 g/cm^2^ with slight variation in several recent studies [20, 21]. Lumbar spine BMD in children varies from approximately 0.6 g/cm^2^ (age 5 years) to 1.0 g/cm^2^ (age 17 years) with much greater variation [18, 19]. A single bone sample would not model the bone density of any childhood vertebra since BMD changes with age. In this series (no BMD measurements), the probable difference between BMD in the femoral neck of elderly patients with hip arthrosis and children’s spines would tend to underestimate the radiation dose needed to satisfactorily visualise the correct placement of pedicle screws in young children because the BMD is considerably lower in children 1–9 years of age. However, this did not cause any problems for the implementation of the optimised protocol. For most children who undergo corrective spinal surgery (10–17 years of age), BMD of the lumbar spine is probably not very different from that of the bone samples used in our study.

A substantial number of pedicle screws are misplaced when navigation is not used. The rate of pedicle perforation varies between 10% and 40% [22–24]. Our own data (unpublished) for the first 30 children where the screws were placed under fluoroscopic guidance and then position-controlled with volumetric imaging indicate that approximately 8% of a total of 424 screws were misplaced (according to the Ohlin classification [24]) and 1% of screws had to be repositioned or removed. Since we started using the cone-beam flat-panel detector scanner in 2008, we have not had any nerve or spinal cord damage caused by misplaced screws. This has been corroborated by several other studies [25–27]. The cost-benefit of navigation is well-documented [5]. It is also well-documented that 3-D techniques are superior to other techniques used for navigation [22, 23]. Some surgeons use freehand techniques relying only on anatomical landmarks. The anatomy of the scoliotic spine in children is highly variable, the pedicles often very small and the deformity often great. Freehand techniques are only feasible for a few very talented and experienced spinal deformity surgeons. Most surgeons would prefer to use some kind of intraoperative imaging guidance. The number of misplaced screws in degenerative adult lumbar spines using freehand techniques was significantly greater than the number of misplaced screws placed following a 3-D scan with subsequent navigation [27]. According to the manufacturers, the radiation dose of one 3-D scan equals that of 35 s of fluoroscopy [7]. Fluoroscopy time has been calculated to be approximately 7–20 s per screw [28–31]. With an average ten screws per child in our case study, this equals 68–193 s of fluoroscopy time per operation, or the equivalent of two to six volumetric scans, representing a higher radiation dose compared to our case study. One also needs to take into account that the pedicle screw misplacement rate is significantly reduced when navigation is based on 3-D imaging [22–24]. The misplacement rate is even higher in children, which further strengthens the case for using volumetric guidance [27].

There are currently few alternatives to the system used in our study [32, 33]. Other available systems are slower and only two to three spinal levels can be imaged simultaneously, resulting in an imaging time of 8–9 min (as compared to 1 min of discontinuation time for the described system). Other low-dose systems like EOS [34, 35] or the system described by Abul-Kassim et al. [24] cannot be used intraoperatively. The EOS system in particular can only be used for standing or sitting patients [34, 35].

Abul-Kasim et al. [36] have also tried to optimise the radiation exposure and image quality of the same system used in our study. Using a completely different model (anthropomorphic adult chest model and porcine spine with pedicle screws), they arrive at almost the same dose settings as we recommend. The pedicle perforation rate in the porcine spine model was higher than the perforation rate simulated in our model, which may explain some difference in observer agreement at low dose.

The cylindrical-equivalent diameter of a body is defined as the diameter of the cylinder that the body would form if laterally compressed into a cylinder of equal cross-sectional area. This definition should not be confused with the definition of patient-equivalent cylinder based on the patient’s weight and height as suggested by The Danish National Board of Health [37]. The equivalent diameter may be calculated as d = 2√ab where a and b are the minor and major diameters of the ellipse. Note that the circumference of the patient-equivalent circle and the elliptic cross-section of the patient are not equal, and can therefore only estimate the diameter of the patient equivalent cylinder with an error of 10–15% depending on the eccentricity of the patient. Given that the difference in the diameter between the patient equivalent phantoms is about 50%, it might be sufficient to use the circumference to calculate the diameter of the patient to select the appropriate protocol.

The phantoms in our study simulated the lumbar anatomy. The results may therefore not adequately reflect the dose reduction achievable in the thorax. Based on the level of scattered radiation in the thorax compared with the lumbar level, we would anticipate the dose in the thorax to be even lower.

The lower-contrast images of the cone-beam scanner are not comparable with a standard CT [4], but in the case of spinal surgery, spatial resolution is of greater concern. Dealing with very high-density material (bone and metal) relative to water, we showed that the needed spatial resolution can be obtained at a fraction of the preset exposure values (Table 2).

The parameters of the optimised protocols were to some extent dictated by limitations of the generator. Even at the lowest possible tube current (10 mA), all phantoms at all voltages above 90 kVp showed acceptable image quality. If the mA were lowered further, a lower patient dose might have been achievable since the same dose at a higher kVp would result in a lower absorbed dose to the patient. Dose to the patient may have been reduced further by using a bow-tie filter typically employed in standard CT scanners [38]. A preliminary study with such a filter has shown that dose may be additionally reduced by a factor of 2. Automated current modulation as proposed by Kalender et al. [39] might also further reduce the dose as would adaptive statistical iterative reconstruction combined with conventional filtered back projection (a further estimated reduction of 30–40% [40]).

## Conclusion

With optimised exposures at 70 kVp/40 mAs for a 1-year-old-equivalent phantom, 70 kVp/80 mAs for a 5-year-old-equivalent phantom and 80 kVp/40 mAs for a 12-year-old-equivalent phantom, radiation doses for intraoperative 3-D imaging with a cone-beam flat-panel detector scanner were reduced at least 89% and could still be used to safely guide the placement of pedicle screws. The effective doses for optimised scans were estimated at approximately 0.5 mSv and were between 91–94,5% lower than the effective dose estimated for the manufacturers' default exposure values.
